# Serum Human Epididymis Protein 4 as a Novel Biomarker in Identifying Patients With Interstitial Lung Disease in Rheumatoid Arthritis

**DOI:** 10.3389/fmed.2021.755268

**Published:** 2021-10-26

**Authors:** Liu Liang, Jiali Chen, Chun Di, Minghua Zhan, Huizhang Bao, Changsheng Xia, Chunhong Fan, Yudong Liu

**Affiliations:** ^1^Department of Clinical Laboratory, Peking University People's Hospital, Beijing, China; ^2^Department of Clinical Laboratory, The Second People's Hospital of Qinzhou, Qinzhou, China; ^3^Department of Rheumatology and Immunology, The Second Xiangya Hospital, Central South University, Changsha, China

**Keywords:** rheumatoid arthritis, human epididymis protein 4, interstitial lung disease, risk stratification, biomarker

## Abstract

**Objective:** Human epididymis protein 4 (HE4) have been implicated in the pulmonary involvements. We aimed to investigate the clinical utility of HE4 in clinical stratification in patients with rheumatoid arthritis (RA).

**Methods:** This study included a discovery cohort comprising 70 RA patients and 64 healthy controls (HCs), and a validation cohort comprising 98 RA patients and 75 HCs. Human epididymis protein 4 were determined by electrochemical luminescence analyzer.

**Results:** The levels of HE4 were significantly elevated in patients with RA compared to HCs. The positive rates of HE4 in patients with RA and HCs were 50.0% and 0, respectively, in the discovery cohort and 53.1 and 1.3%, respectively, in the validation cohort. When RA patients were subgrouped according to HE4 status, HE4-positive group displayed higher prevalence of interstitial lung disease (ILD) compared to HE4-negative group (28.6 vs. 11.4% in discovery cohort and 57.7 vs. 8.7% in the validation cohort). A positive correlation between the levels of HE4 with the degree of lung impairment was identified. Receiver operating curve (ROC) analysis revealed an optimal cut-off value of 104.3 pmol/L in HE4 for distinguishing RA-ILD from RA-non ILD with the areas under the curve (AUC) of 0.790. Multivariate logistic regression analysis illustrated that high levels of HE4 independently identified patients with RA-ILD (OR, 9.080*, p* < 0.001).

**Conclusion:** Our findings showed a novel role of HE4 in RA risk stratification, suggest that introducing HE4 to the current RA test panel may serve as an indicator in identifying RA patients for further RA-ILD workups, such as high-resolution computed tomography (HRCT).

## Introduction

Rheumatoid arthritis (RA) is a chronic and progressive autoimmune joint disorder characterized by synovial inflammation, joint destruction, and various extra-articular manifestations (EAMs) (1–3). Pulmonary complications, particularly interstitial lung disease (ILD), represent an important extra-articular feature of various EAMs and a major cause of mortality in patients with RA (2–5). A recent study showed that 72% patients with RA-ILD had an inpatient admission and 76% had an emergency room visit (6). Further, clinically significant RA-ILD accounts for 10% of the RA population, and is associated with shortened survival and more severe underlying disease (7). To date, there are few treatments that are proven to be effective in the treatment of RA-ILD, and biomarkers that can predict RA patients at risk for ILD are in great need.

Human epididymis protein 4 (HE4), a human epididymis-specific protein, has been widely utilized in clinical practice as a tumor marker in identifying patients at risk of ovarian cancer (8). Human epididymis protein 4 is expressed in multiple tissues in the oral cavity, the respiratory tracts as well as in renal tubular epithelial cells (9). Previous studies have shown that serum HE4 levels were significantly elevated in patients with systemic sclerosis (SSc)-ILD (10). Further, high levels of HE4 can stratify patients into SSc-ILD subsets (10). We also showed that HE4 can identify primary Sjögren's syndrome (pSS) patients at risk of pulmonary/renal involvements (11). Taken together, these findings suggest that HE4 may have a diagnostic potential in disease stratification in patients with autoimmune diseases. In this study, we aimed to investigate the clinical utility of HE4 in the diagnosis and disease stratification in RA.

## Materials and Methods

### Subjects

To evaluate the clinical utility of HE4 in RA patients, we recruited two cohorts of RA patients, including the discovery cohort and the validation cohort (Table 1). The discovery cohort retrospectively collected the clinical data of 70 consecutive RA inpatients who were tested for the female-tumor biomarker screening panel from Jan. 2020 to Sep. 2020 and 64 consecutive healthy controls (HCs) who went to Peking University People's Hospital (PKUPH) for annual physical examination and were tested for the female-tumor biomarker screening panel between Jan. 2020 and Oct. 2020. To verify the clinical relevance of HE4 for RA patients, we prospectively recruited 98 RA inpatients and 75 HCs and regarded those patients as the validation cohort. All RA patients were diagnosed according to 2010 American College of Rheumatology (ACR)/European League Against Rheumatism classification criteria (12). Rheumatoid Arthritis patients with cancer or history of malignant neoplasm were excluded. Healthy controls who displayed abnormality in blood differential tests, biochemistry profile tests, autoantibody profile tests or had history of systemic diseases, neoplastic, and autoimmune/autoinflammatory diseases were excluded. The study protocol was reviewed and approved by the Ethical Committee of PKUPH (Protocol number: 2019PHB244). Informed consents of discovery cohort were waived for those patients in the discovery cohort. However, all participants in the validation cohort gave written informed consent.

**Table 1:** Demographic and clinical characteristics between patients with rheumatoid arthritis (RA) and healthy controls (HCs).

	**Discovery cohort**			**Validation cohort**		
	**RA**	**HC**	***p*-value**	**RA**	**HC**	***p*-value**
	**(*n* = 70)**	**(*n* = 64)**		**(*n* = 98)**	**(*n* = 75)**	
Age, years	62 (53, 70)	40 (31, 49)	<0.001	64 (55, 71)	60 (52, 69)	0.154
Gender, female	70 (100.0)	64 (100.0)	—	77 (78.6)	55 (73.3)	0.259
Smoking status	2 (2.9)	—	—	5 (5.1)	—	—
Duration, years	8.5 (3.0, 17.0)			9.5 (2.9, 19.0)		
SJC28	2 (0, 8)			3.5 (0, 10)		
TJC28	4 (1, 14)			8 (1,16)		
DAS28-ESR	5.4 (3.9, 7.2)			5.2 (3.7, 6.8)		
Anti-CCP, positive	60 (85.7)			87 (88.8)		
RF, positive	52 (74.3)			79 (80.6)		
Interstitial lung disease	14 (20.0)			34 (34.7)		
ESR, mm/h	39 (18, 81)			39 (15, 74)		
CRP, mg/L	13.5 (2.4, 31.4)			14.6 (1.3, 32.2)		
Medications						
NSAIDs	20 (28.6)			16 (16.3)		
Steroids	18 (25.7)			32 (32.7)		
cDMARDs	52 (74.3)			60 (61.2)		
bDMARDs	10 (14.3)			11 (11.2)		

*Data are presented as median (interquartile range) and n (%). Anti-CCP, anti-cyclic citrullinated peptide antibody; bDMARDs, biological disease modifying anti-rheumatic drugs; cDMARDs, conventional disease modifying anti-rheumatic drugs; CRP, C-reactive protein; DAS28-ESR, disease activity score-ESR; ESR, erythrocyte sedimentation rate; ILD, interstitial lung disease; NSAIDs, non-steroid anti-inflammatory drugs; RF, rheumatoid factor; SJC28, swollen joint counts in 28 joints; TJC28, tender joint counts in 28 joints*.

### Data Collection

Demographic features and clinical and laboratory findings, including age, disease duration, swollen joint count in 28 joints (SJC28), tender joint count in 28 joints (TJC28), erythrocyte sedimentation rate (ESR), C-reactive protein (CRP), rheumatoid factor (RF), anti-CCP antibodies, EAMs, treatments and medical history, were collected from the medical database of PKUPH. Disease Activity Score (DAS28) was assessed as previous described (13, 14).

### Serum Tumor Biomarkers Determination

All the tumor biomarkers in the female-tumor biomarker screening panel, including alpha fetoprotein (AFP), carcinoembryonic antigen (CEA), neuron-specific enolase (NSE), cytokeratin 19 fragment antigen 21-1 (CYFRA21-1), cancer antigen 125 (CA125), CA15-3, CA19-9, progastrin-releasing peptide (ProGRP), squamous cell carcinoma (SCC), and HE4 were determined by Roche Cobas electrochemical luminescence analyzer (Hoffmann-La Roche AG., Basel, Switzerland), according to the manufacturer's instructions. The cut-off values for positivity of these tumor markers were determined based on the manufacturer's recommendations.

### Assessment of Interstitial Lung Disease

The assessment of ILD was performed by two pulmonologists and two radiologists with more than 10 years of thoracic imaging experience, mainly based on the symptoms and respective abnormalities suggestive of ILD in high-resolution computed tomography (HRCT) and pulmonary function tests (PFTs). The RA-ILD was assessed semi-quantitatively based on the HRCT scans, and any indeterminate ILD were excluded from the analyses. Pulmonary function tests were determined as forced vital capacity (FVC), the median forced expiratory volume in 1 second (FEV1), and diffusing capacity of the lung for carbon monoxide (DLco). Forced vital capacity, FEV1, and DLco were presented as a percentage of the predicted values for the patient's age, sex, and height, as previously described (15). Abnormalities of PFTs were defined as predicted values of FVC <80% and DLco <70% (16).

### Statistical Analysis

Continuous variables were presented as mean ± standard deviation (SD) for normal distribution or median (interquartile range, IQR) for abnormal distribution. Categorical variables were shown as numbers (percentages) of the total samples. The statistical significance between groups was assessed using the Mann-Whitney U-test, Student *t*-test, Chi-square (χ^2^)-test, where it was applicable. Spearman's correlation test was used to determine the relationships between HE4 and clinical parameters. The receiver operating characteristic (ROC) curve was generated to evaluate the sensitivity, specificity and areas under the ROC curve (AUC) with the 95% confidence interval (95% CI). The optimal cutoff value for predicting the incidence of ILD was identified by calculating the Youden index. Multivariate Logistic regression analyses were used to determine the risk factors for ILD. Once a univariate statistic was generated, the multivariate model was then built using a forward selection procedure. Variables with a *p*-value of <0.1 in the univariate analysis were first considered as candidates for the multivariate model, then variables with a *p*-value of <0.05 were used in the final model, and odds ratios (ORs) were calculated with 95% CI. Data analyses were calculated using SPSS 20.0 statistical software package (SPSS Inc., Chicago, Illinois, USA) or GraphPad Prism 8 (GraphPad Software Inc.). A significant difference was defined as *p* <0.05.

## Results

### Characteristics of Patients With RA in the Two Cohort

Detailed clinical and laboratory characteristics of the two cohort are presented in Table 1. The median duration of RA patients was 8.5 years for the discovery cohort and 9.5 years for the validation cohort. The median DAS28 was 5.4 (IQR 3.9, 7.2) for RA patients in the discovery cohort and 5.2 (IQR 3.7, 6.8) for RA patients in the validation cohort. Anti-CCP antibodies were positive in 85.7% of RA patients in the discovery cohort and 88.8% of RA patients in the validation cohort. Rheumatoid factor was positive in 74.3% of RA patients in the discovery cohort and 80.6% of RA patients in the validation cohort. The prevalence of RA-ILD was 20.0% in the discovery cohort and 34.7% in the validation cohort, respectively.

### The Levels of HE4 Were Significantly Elevated in Patients With RA

The cut-off values for positivity of all tumor markers in the female-tumor biomarker screening panel are listed in Table 2. Compared to other tumor markers, HE4 displayed the highest positive rates in RA patients (50.0%), followed by CYFRA21-1 (21.4%) and CA125 (20.0%) (Table 2). No HCs were detected positive for HE4. Of interest, the prevalence of HE4 in RA patients <60 years old, RA patients aged between 60 and 70 years and RA patients over 70 years old were 22.6, 66.7, and 77.8%, respectively.

**Table 2:** Levels of positive tumor markers between patients with rheumatoid arthritis (RA) and healthy controls (HCs).

**Positive biomarkers**	**Cut-off value**	**Discovery cohort**			**Validation cohort**		
		**RA**	**HC**	***p*-value**	**RA**	**HC**	***p*-value**
		**(*n* = 70)**	**(*n* = 64)**		**(*n* = 98)**	**(*n* = 75)**	
CEA, *n* (%)	4.7 ng/ml	9 (12.9)	0 (0.0)	0.003			
AFP, *n* (%)	26.4 ng/ml	1 (1.4)	3 (4.7)	0.348			
CA19-9, *n* (%)	39 U/ml	8 (11.4)	1 (1.6)	0.035			
CA125, *n* (%)	35 U/ml	14 (20.0)	2 (3.1)	0.003			
CA15-3, *n* (%)	26.4 U/ml	4 (5.7)	0 (0.0)	0.121			
CYFRA21-1, *n* (%)	3.3 ng/ml	15 (21.4)	8 (12.5)	0.251			
NSE, *n* (%)	16.3 ng/ml	2 (2.9)	7 (10.9)	0.086			
ProGRP, *n* (%)	68.3 pg/ml	10 (14.3)	0 (0.0)	0.002			
SCC, *n* (%)	2.7 ng/ml	4 (5.7)	2 (3.1)	0.682			
HE4, *n* (%)	76.2 pmol/L (<60 years)	7/31 (22.6)	0/61 (0.0)	<0.001	13/34 (38.2)	0/37 (0.0)	<0.001
	82.9 pmol/L (60–69 years)	14/21 (66.7)	0/1 (0.0)	<0.001	18/31 (58.1)	1/21 (4.8)	0.002
	104 pmol/L (≥70 years)	14/18 (77.8)	0/2 (0.0)	<0.001	21/33 (63.6)	0/17 (0.0)	<0.001
	Overall	35 (50.0)	0 (0.0)	<0.001	52 (53.1)	1 (1.3)	<0.001

*Data are presented as n (%). Cut-off value was determined based on the manufacturer's recommendations. AFP, alpha fetoprotein; CA125, cancer antigen 125; CA15-3, cancer antigen 15-3; CA19-9, cancer antigen 19-9; CEA, carcino-embryonic antigen; CYFRA21-1, cytokeratin 19 fragment antigen 21-1; HE4, human epididymis protein 4; NSE, neuron-specific enolase; ProGRP, progastrin-releasing peptide; SCC, squamous cell carcinoma antigen*.

Since HE4 was the most significant marker among all the tumor markers in female-tumor biomarker screening panel, we next focus the clinical performance of HE4 in RA. We first verified these results in the validation cohort. The positive rate of HE4 was 53.1% in this cohort, which was similar to the discovery cohort (Table 2). A similar trend was also observed in RA patients in the validation cohort.

**Figure 1:**
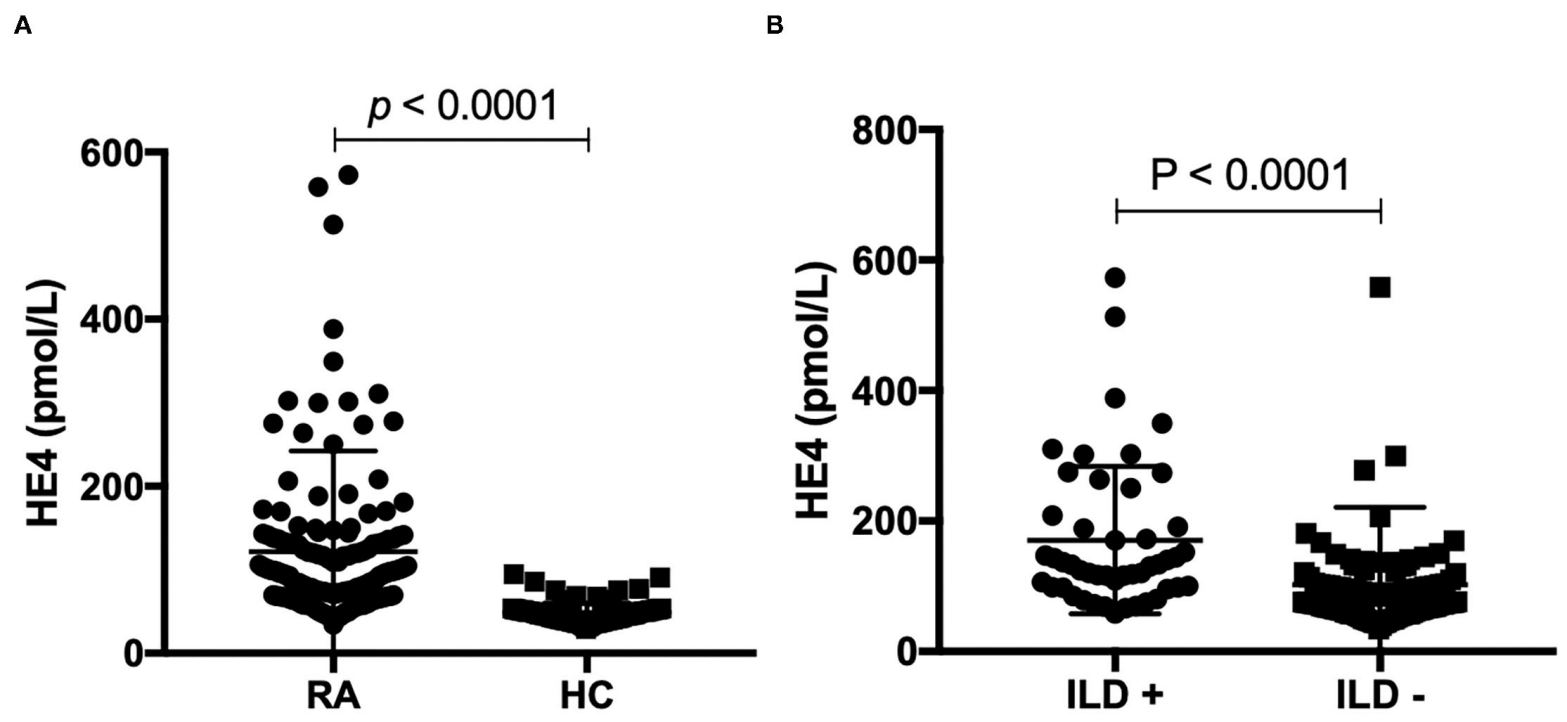
**(A)** Levels of human epididymis protein 4 (HE4) were elevated in patients with rheumatoid arthritis (RA) (*n* = 168) compared to healthy controls (HCs) (*n* = 139). Associations between levels of HE4 with **(B)** Levels of human epididymis protein 4 (HE4) were elevated in rheumatoid arthritis (RA) patients with interstitial lung disease (ILD) (*n* = 48) compared to RA patients without ILD (*n* = 120).

**Figure 2:**
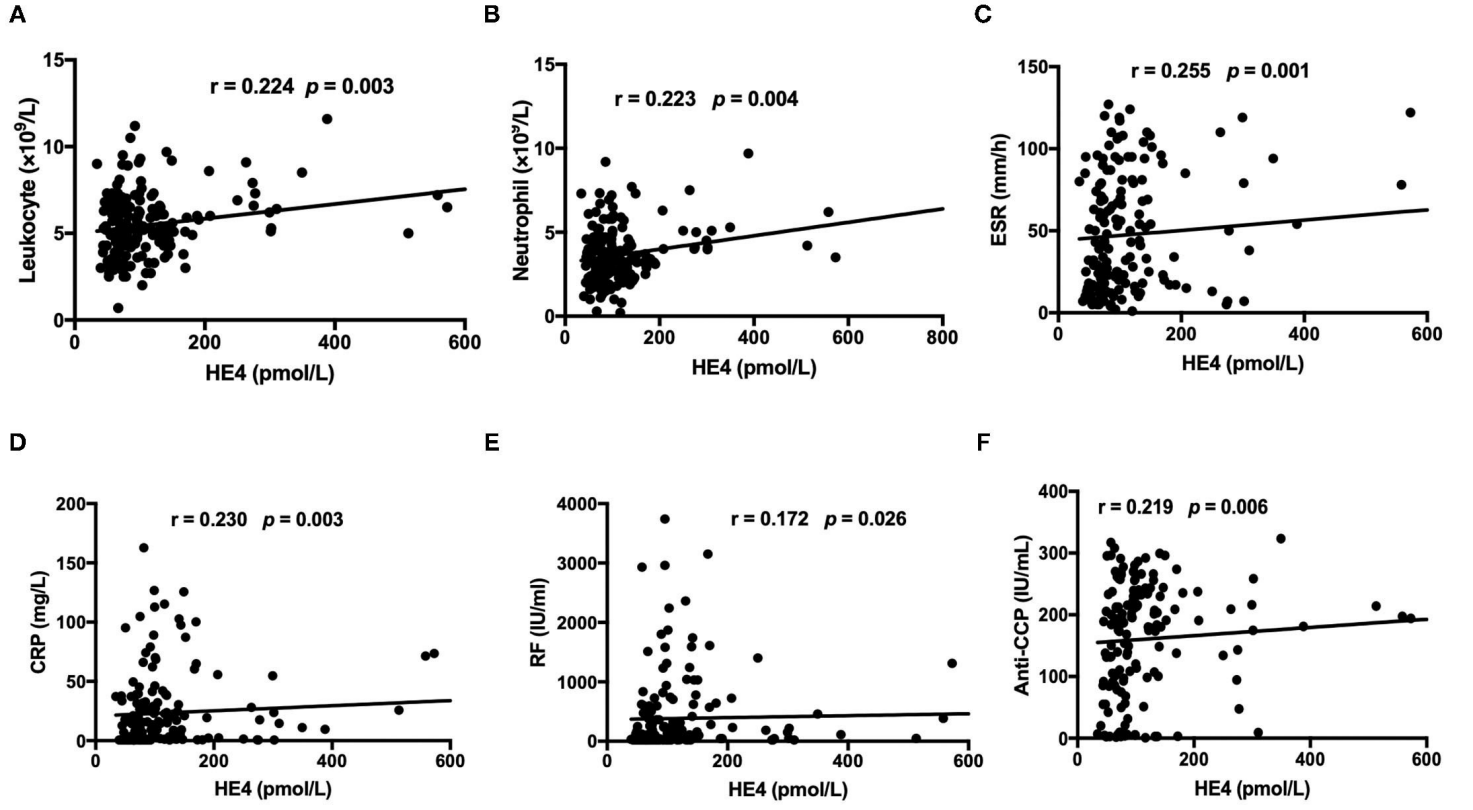
Associations between levels of HE4 with **(A)** total leukocyte count, **(B)** total neutrophil count, **(C)** erythrocyte sedimentation rate (ESR), **(D)** C-reactive Protein (CRP), **(E)** Rheumatoid factor, and **(F)** anti-CCP antibodies (*n* = 168).

The levels of HE4 were significantly elevated in patients with RA compared to those in HCs (Figure 1A). Further, patients with RA-ILD exhibited significantly higher levels of HE4 compared to patients without RA-ILD (*p* < 0.0001) (Figure 1B). The levels of HE4 were significantly associated with total leukocytes number (*p* = 0.003), total neutrophil number (*p* = 0.003), ESR (*p* = 0.001), CRP (*p* = 0.003), RF (*p* = 0.026), and anti-CCP (*p* = 0.006) (Figures 2A–F).

### Characteristics of Patients With RA According to HE4 Levels

**Figure 3:**
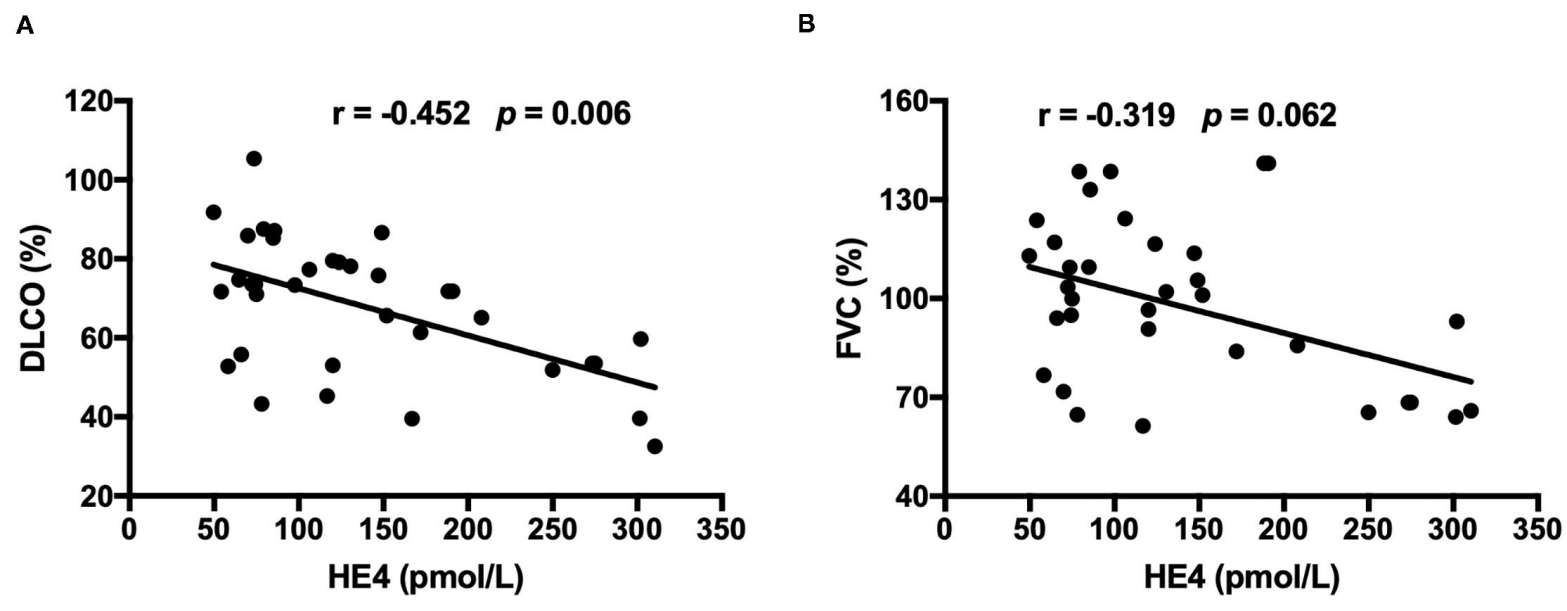
**(A)** Associations between levels of HE4 and diffusing capacity of the lung for carbon monoxide (DLco) which was presented as a percentage of the predicted values for the patient's age, sex, and height (*n* = 35). **(B)** Associations between levels of HE4 and forced vital capacity (FVC), which was presented as a percentage of the predicted values for the patient's age, sex, and height (*n* = 35).

Clinical and laboratory characteristics were compared between HE4-positive RA patients and HE4-negative RA patients. Patients in the HE4-positive group displayed similar disease duration and DAS28-ESR (Table 3). Further, HE4-positive patients showed similar laboratory characteristics, including RF positivity, anti-CCP positivity, levels of C3, levels of C4, and levels of IgG (Table 3). Of interest, the prevalence of RA-ILD in patients positive for HE4 was twice as much as that in patients negative for HE4 (28.6 vs. 11.4%, *p* = 0.073) (Table 3). The higher incidence of RA-ILD in HE4-positive RA was also confirmed in the validation cohort (57.7 vs. 8.7%, *p* < 0.001). Consistent with higher levels of HE4 in patients with RA-ILD, the levels of HE4 displayed a significantly negative correlation with DLco% (*r* = −0.452, *p* = 0.006) (Figure 3A). In addition, a negative correlation between the levels of HE4 and FVC% (*r* = −0.319, *p* = 0.062) was noticed (Figure 3B). In contrast, no significant correlations between RF or anti-CCP and DLco% or FVC% were identified (Table 4).

**Table 3:** Clinical characteristics of patients with rheumatoid arthritis (RA) according to HE4 status*.

	**Discovery cohort**			**Validation cohort**		
	**HE4+**	**HE4–**	***p*-value**	**HE4+**	**HE4–**	***p*-value**
	**(*n* = 35)**	**(*n* = 35)**		**(*n* = 52)**	**(*n* = 46)**	
Age, years, median (IQR)	69 (62, 77)	56 (50, 62)	<0.001	67 (56, 74)	61 (54, 67)	0.427
Gender, female, *n* (%)	35 (100.0)	35 (100.0)	–	38 (73.1)	39 (84.8)	0.159
Smoking status	0 (0.0)	2 (5.7)	0.493	5 (9.6)	0 (0.0)	0.058
Duration, years, median (IQR)	9 (3, 18)	8 (3, 16)	0.306	12 (9, 18)	8 (3, 21)	0.210
SJC28, median (IQR)	2 (1, 10)	2 (0, 6)	0.245	2 (0, 5)	7 (2, 11)	0.008
TJC28, median (IQR)	4 (1, 4)	4 (2, 12)	0.831	6 (1, 17)	8 (3, 16)	0.313
ESR, mm/h, median (IQR)	66 (25, 93)	39 (20, 62)	0.038	53 (24, 80)	43 (16, 76)	0.230
CRP, mg/L, median (IQR)	14.6 (4.3, 42.0)	10.7 (2.1, 29.1)	0.180	16.2 (5.5, 28.6)	5.6 (0.6, 37.5)	0.021
DAS28-ESR, median (IQR)	5.77 (4.61, 8.04)	5.54 (4.11, 6.85)	0.226	5.84 (3.81, 6.85)	6.58 (5.24, 8.24)	0.196
RF, positive, *n* (%)	27 (77.1)	25 (71.4)	0.584	46 (88.5)	33 (71.7)	0.037
RF, positive, IU/ml	254.0 (79.7, 729.5)	29.9 (22.8, 138.4)	0.004	201.0 (44.1, 960.3)	63.8 (20.0, 298.0)	0.008
Anti-CCP, positive, *n* (%)	31 (88.6)	29 (82.9)	0.495	46 (88.5)	41 (89.1)	0.917
Anti-CCP, positive, U/ml	225.6 (173.4, 257.4)	167.3 (64.2, 229.5)	0.734	199.4 (147.3, 234.6)	136.4 (83.0, 196.5)	0.004
NLR, median (IQR)	3.0 (2.04, 4.46)	2.71 (1.79, 3.97)	0.262	2.54 (1.87, 4.56)	2.56 (1.86, 3.65)	0.184
Platelets, 10^9^/L, median (IQR)	281 (213, 339)	280 (220, 320)	0.944	225 (178, 296)	242 (198, 310)	0.438
Hemoglobin, g/L, median (IQR)	103 (95, 116)	114 (101, 121)	0.165	105 (94, 121)	115 (97, 132)	0.300
C3, g/L, median (IQR)	0.99 (0.86, 1.18)	0.99 (0.89, 1.19)	0.874	0.92 (0.80, 1.03)	1.09 (0.88, 1.21)	0.017
C4, g/L, median (IQR)	0.19 (0.14, 0.25)	0.21 (0.17, 0.26)	0.585	0.22 (0.17, 0.26)	0.21 (0.18, 0.25)	0.078
IgG, g/L, median (IQR)	13.3 (9.7, 15.9)	13.9 (11.4, 19.0)	0.224	12.9 (8.9, 16.9)	15.1 (11.1, 17.9)	0.298
Interstitial lung disease, *n* (%)	10 (28.6)	4 (11.4)	0.073	30 (57.7)	4 (8.7)	<0.001
Leukocytopenia, *n* (%)	4 (11.4)	6 (17.1)	0.495	3 (5.8)	10 (21.7)	0.020
Anemia, *n* (%)	22 (62.9)	20 (57.1)	0.626	32 (61.5)	20 (43.5)	0.115
Sjögren's syndrome, *n* (%)	1 (2.9)	0 (0.0)	1.000	21 (40.4)	17 (37.0)	0.728
Medications						
NSAIDs, *n* (%)	13 (37.1)	7 (20.0)	0.112	8 (15.4)	8 (17.4)	0.530
Steroids, *n* (%)	11 (31.4)	7 (20.0)	0.274	18 (34.6)	14 (30.4)	0.802
cDMARDs, *n* (%)	27 (77.1)	25 (71.4)	0.584	33 (63.5)	27 (58.7)	0.709
bDMARDs, *n* (%)	3 (8.6)	7 (20.0)	0.172	5 (9.6)	6 (13.0)	0.517

*The reference range of human epididymis protein 4 (HE4) was determined based on the manufacturer's recommendations. Data are presented as median (Interquartile Range) and n (%). Statistical significance was determined by Mann-Whitney U test and Chi-square (χ2) test. IQR, interquartile range; Anti-CCP, anti-cyclic citrullinated peptide antibody; bDMARDs, biological disease modifying anti-rheumatic drugs; cDMARDs, conventional disease modifying anti-rheumatic drugs; CRP, C-reactive protein; DAS28-ESR, disease activity score-ESR; ESR, erythrocyte sedimentation rate; ILD, interstitial lung disease; NLR, neutrophil/lymphocyte ratio; NSAIDs, non-steroid anti-inflammatory drugs; RF, rheumatoid factor; SJC28, swollen joint counts in 28 joints; TJC28, tender joint counts in 28 joints.

**Table 4:** Associations between levels of rheumatoid factor (RF) or anti-cyclic citrullinated peptide antibody (Anti-CCP) and diffusing capacity of the lung for carbon monoxide (DLco) or forced vital capacity (FVC) in rheumatoid arthritis (RA) patients with interstitial lung disease (ILD)^*^.

	**Anti-CCP**		**RF**	
	**r**	***p-*value**	**r**	***p-*value**
FVC%	−0.242	0.198	0.059	0.756
DLCO%	−0.059	0.756	0.094	0.621

* *DLco or FVC was presented as a percentage of the predicted values for the patient's age, sex, and height. r was determined by Spearman's correlation test*.

### Diagnostic Potential of HE4 in RA-ILD

**Figure 4:**
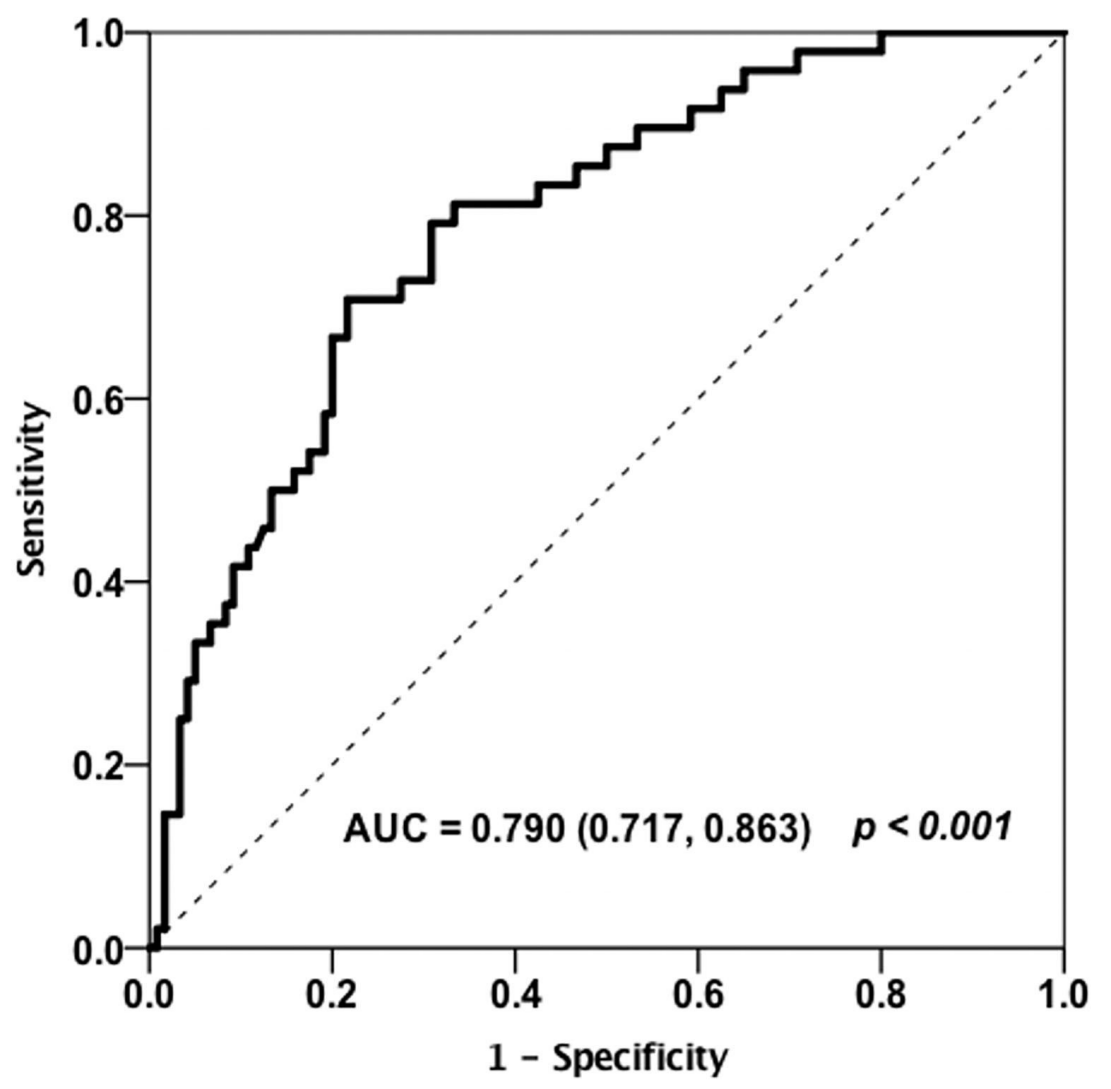
Receiver operating curve (ROC) analysis on the clinical performance of HE4 in identifying patients with RA-ILD (*n* = 168).

Receiver operating curve (ROC) analysis was utilized to characterize the clinical performance of HE4 in identifying patients with RA-ILD (Figure 4). Based on Youden index, the optimal cut-off value of HE4 for distinguishing RA-ILD from RA-non ILD was 104.3 pmol/L with an area under the curve (AUC) of 0.790 and a sensitivity and a specificity of 70.8 and 77.5%, respectively. In addition, ROC analysis was also performed to calculate the cut-off values of high-CCP and high-RF, and the optimal levels were 133.14 U/ml and 170.5 IU/ml, respectively. These cut-off values were further used to identify which RA patients are at risk of having ILD (Table 5). Univariable analysis showed that male gender (*p* = 0.002), old age (*p* = 0.012), and patients positive for HE4 (*p* < 0.001) were risk factor in predicting RA-ILD. In contrast, RA-non ILD patients tended to show higher SJC28 (*p* = 0.024) and TJC28 (*p* = 0.002) compare to patients with RA-ILD. Besides, patients with ILD had higher percentages of high-RF and high-anti-CCP than those without, but the differences were insignificant. To further assess the independent predictors for developing RA-ILD, a multivariate logistic regression analysis was performed. Of note, female gender (OR, 0.275; 95%CI, 0.085–0.893; *p* = 0.032), DAS28-ESR (OR, 0.821; 95%CI, 0.690–0.976; *p* = 0.026), and high levels of HE4 (OR, 9.080; 95%CI, 3.481–23.682; *p* < 0.001) persisted as independent risk factors for predicting RA-ILD (Table 5).

**Table 5:** Univariate and multivariate analyses for risk factors of rheumatoid arthritis (RA)-interstitial lung disease (ILD).

**Variables**	**Univariate analysis**			**Multivariate analysis**		
	**ILD+ (*n* = 48)**	**ILD– (*n* = 120)**	***p*-value**	**ORs**	**95%CI**	***p*-value**
Gender, female	36 (75.0)	111 (92.5)	0.002	0.275	0.085–0.893	0.032
Age, year	67 (57, 73)	62 (53, 70)	0.012	1.002	0.961–1.045	0.992
Smoking status	4 (8.3)	3 (2.5)	0.104			
Disease duration, year	9 (4, 15)	9 (3, 20)	0.938			
SJC28	1 (0, 4)	2 (0, 8)	0.024			
TJC28	1 (0, 8)	6 (1, 14)	0.002			
ESR≥20 mm/h	31 (64.6)	86 (71.7)	0.367			
CRP≥8.0 mg/L	27 (56.3)	70 (58.3)	0.805			
DAS28-ESR	4.74 (3.26, 6.10)	5.46 (3.99, 7.27)	0.035	0.821	0.690-0.976	0.026
High-RF, positive	23 (47.9)	42 (35.0)	0.120			
High-anti-CCP antibody, positive	34 (70.8)	67 (55.8)	0.073	1.410	0.602–3.301	0.429
Leukopenia	5 (10.4)	18 (15.0)	0.435			
Anemia	23 (47.9)	71 (59.2)	0.185			
Hyperglobulinemia	10 (20.8)	18 (15.0)	0.359			
High HE4, positive	34 (70.8)	27 (22.5)	<0.001	9.080	3.481–23.682	<0.001
Sjögren's syndrome	11 (22.9)	28 (23.3)	0.954			
NSAIDs	8 (16.7)	28 (23.3)	0.341			
Steroids	18 (37.5)	32 (26.7)	0.165			
cDMARDs	40 (83.3)	92 (76.7)	0.341			
bDMARDs	5 (10.4)	16 (13.3)	0.606			

*Data are presented as median (Interquartile Range) and n (%). Statistical significance was determined by Mann-Whitney U test, Chi-square (χ2) test and Logistic regression analysis. Leukopenia was defined as white blood cell count <4,000/mm^3^. Anemia was defined as hemoglobin levels <110 g/L for women. Hyperglobulinemia was defined as immunoglobulin G (IgG) level >16.8 g/L. High HE4 was defined as HE4 levels > 104.25 pmol/L based on Youden index. Anti-CCP, anti-cyclic citrullinated peptide antibody; bDMARDs, biological disease modifying anti-rheumatic drugs; cDMARDs, conventional disease modifying anti-rheumatic drugs; CRP, C-reactive protein; DAS28, disease activity score; ESR, erythrocyte sedimentation rate; NSAIDs, non-steroid anti-inflammatory drugs; RF, rheumatoid factor; SJC, swollen joint counts in 28 joints; TJC, tender joint counts in 28 joints*.

## Discussion

Human epididymis protein 4 has been widely utilized as an effective biomarker in the diagnosis and follow-up of patients with ovarian cancer. Of interest, accumulating evidence suggest that HE4 may also has diagnostic potential in other clinical settings, including lung adenocarcinomas (17), renal fibrosis (18), cystic fibrosis (19), as well as autoimmune-related organ involvements, such as SSc-ILD (10) and pSS-related pulmonary/renal involvements (11). In this study, we extended the clinical application of HE4 into the diagnosis of RA. We found that the levels of HE4 were significantly elevated in patients with RA, particularly in patients with RA-ILD. Further, we showed that high levels of HE4 were an independent factor for identifying patients with RA-ILD. Given that biomarkers predicting RA patients at risk for ILD are currently lacking, our findings thus represent an important endeavor in risk stratification and clinical subset identification in patients with RA.

In this study, we found that the levels of HE4 were elevated in more than half of RA patients. When we further subgrouped RA patients according to HE4 status, we found that HE4-positive RA group had a higher percentage of RA-ILD compared to HE4-negative RA group. This phenotype was also verified in a separate RA cohort. Recently, Zhang et al. reported that serum HE4 levels were significantly increased in patients with SSc-ILD compared to SSc-non-ILD, which was consistent with our findings (10). Further, we found that high levels of HE4 were independently associated with the presence of RA-ILD in a multivariate logistic regression analysis. Consistent with our results, Nishiyama et al. also showed that HE4 was a new biomarker to predict the prognosis of progressive fibrosing ILD (20). Taken together, these findings support a role of HE4 in risk stratification of ILD.

In this study, we showed a positive correlation between the levels of HE4 with the degree of lung impairment. Human epididymis protein 4 has been shown to be expressed in respiratory epithelium (9) and increased expression of HE4 was identified in lung biopsy from patients with cystic fibrosis (CF) (17). A recent study found that elevated levels of HE4 was positively associated the degree of pulmonary dysfunction in patients with CF (19), which was consistent with our study. Further, the levels of HE4 inversely correlated with lung function improvement in CF patients after treatment, suggesting a diagnostic potential of HE4 in routine clinical and laboratory follow-up of CF treatment (21). Although it remains unclear whether HE4 is implicated in the pathogenesis of RA-ILD, LeBleu et al. have showed that HE4 can suppress the activity of multiple proteases, including serine proteases and matrix metalloproteinases, and specifically inhibits their capacity to degrade type I collagen, thereby promoting the development of kidney fibrosis (18). Further studies are needed to define the functional relevance of HE4 in the pathogenesis of RA-ILD.

Of interest, 25 (72%) RA patients in the discovery cohort and 22 (42%) RA patient in the validation cohort were positive for HE4, but did not have ILD, suggesting other RA-related factors may influenced the levels of HE4. Further studies investigating how the levels of HE4 were modulated in the context of RA will be of great importance. In addition, Krebs von den Lungen-6 (KL-6) has been proposed as a potential biomarker in the diagnosis of ILD (22). It will be of great interest to assess whether combination of KL-6 with HE4 can improve the diagnostic value of each single biomarker.

Our study has a number of notable strengths. To the best of our knowledge, our study represents the first study investigating the clinical performance of HE4 in risk stratification of RA. Our findings thus expand our understanding of the clinical utility of HE4 in clinical practice, especially in rheumatoid diseases, such as RA. It should be noted, however, that our study has several limitations. First, it was a single-center study with a small RA cohort. Second, most participants of our study were female, which may result in analysis bias, as RA-ILD are more frequent in male patients. Third, patients of this study had long disease durations, and the levels of HE4 can't represent the onset status. Fourth, longitudinal examination of HE4 in patients with RA was missing. Further multi-center studies with a larger cohort comprising more male patients will be needed to corroborate our findings.

In summary, our findings showed a novel role of HE4 in RA risk stratification, suggesting that introducing HE4 to the current RA test panel (i.e., anti-CCP and RF) may provide additional diagnostic value to the current clinically available assays, especially in identifying RA patients for further RA-ILD workups, such as HRCT. Since RA-ILD represents a major complication responsible for morbidity and mortality in RA, this simple and highly reproducible biomarker, which has been already available in routine clinical practice in some countries, would be of great importance to risk-stratify RA patients for the performance of HRCT.

## Data Availability Statement

The raw data supporting the conclusions of this article will be made available by the authors, without undue reservation.

## Ethics Statement

The studies involving human participants were reviewed and approved by the Ethical Committee of PKUPH (Protocol number: 2019PHB244). The patients/participants provided their written informed consent to participate in this study.

## Author Contributions

LL, JC, and CD: experiments, data acquisition, and data analysis. MZ, HB, CX, and CF: experiments and data acquisition. YL: study design, data analysis, and manuscript preparation. All authors contributed to the article and approved the submitted version.

## Funding

This work was supported in part by grants from National Natural Science Foundation of China, grant no. 81971521.

## Conflict of Interest

The authors declare that the research was conducted in the absence of any commercial or financial relationships that could be construed as a potential conflict of interest.

## Publisher's Note

All claims expressed in this article are solely those of the authors and do not necessarily represent those of their affiliated organizations, or those of the publisher, the editors and the reviewers. Any product that may be evaluated in this article, or claim that may be made by its manufacturer, is not guaranteed or endorsed by the publisher.
